# New and underutilized uses of umbilical cord blood in neonatal care

**DOI:** 10.1186/s40748-015-0017-2

**Published:** 2015-06-16

**Authors:** Patrick D. Carroll, Robert D. Christensen

**Affiliations:** Women and Newborn’s Program, Intermountain Healthcare, Salt Lake City, UT USA; Neonatal Services, Dixie Regional Medical Center, St. George, UT USA; Division of Neonatology and Division of Hematology/Oncology, Department of Pediatrics, University of Utah School of Medicine and Primary Children’s Hospital, Salt Lake City, UT USA

**Keywords:** Neonate, Cord blood, Phlebotomy, Transfusion, Placenta, Delayed cord clamping, Cord milking

## Abstract

**Background:**

In an era increasingly focused on quality improvement and cost containment, more emphasis is being placed on wiser utilization of medical care resources. One underutilized resource in early neonatal care is umbilical cord blood.

**Findings:**

Umbilical cord blood can be utilized for admission laboratory studies in neonates thereby avoiding a significant phlebotomy event in the first minutes to hours of life. Additionally, umbilical cord blood can also be safely “transfused” into the neonate via delayed cord clamping or milking of the umbilical cord. This has been demonstrated to be particularly beneficial in premature infants by decreasing the rate of intraventricular hemorrhage. Delayed cord clamping has been formally endorsed by a number of medical societies, however it has not yet been universally adopted by obstetricians and neonatologists.

**Conclusions:**

Both uses of umbilical cord blood for neonatal admission laboratory testing and delayed cord clamping/milking of the umbilical cord have resulted in decreased transfusion rates as well as other outcomes reviewed herein.

## Introduction

In an era increasingly focused on quality improvement and cost containment, more emphasis is being placed on wiser utilization of medical care resources. Emphasis has included aspects such as consistency in the use of medical interventions, creating evidence-based “bundles” of care, and process standardization. One underutilized resource in early neonatal care is the use of umbilical cord blood. This review will present proposed uses of this resource in the minutes immediately following delivery. Specific uses will include utilizing otherwise discarded umbilical cord blood for all admission laboratory blood studies, and delayed cord clamping (DCC) or milking of the umbilical cord (MUC). We will not review the use of umbilical cord blood for autologous transfusion, umbilical cord blood banking, or cord blood mesenchymal stem cells for neonatal therapy in this manuscript.

## Findings

### Drawing all initial laboratory blood tests of very low birth weight (VLBW) infants using fetal blood in the cord/placenta, thereby drawing no blood initially on the VLBW neonate

Blood tests are required to care for critically ill neonatal intensive care unit (NICU) patients, including VLBW infants. VLBW infants typically have greater phlebotomy blood loss, per kilogram body weight, on the first day of life than any other day during their hospitalization. In one study the mean phlebotomy loss on the first day of life was >10 mL/kg [1]. Laboratory studies performed on admission can require up to 10 % of an extremely low birth weight (ELBW) neonate’s circulating blood volume. Obtaining admission laboratory studies from the otherwise discarded fetal blood in the umbilical cord or placenta is an alternative to drawing admission laboratory studies directly from the infant. In the following paragraphs we will review the data on using umbilical cord blood for complete blood count (CBC), blood culture, blood type, antibody screen, newborn metabolic screening, and genetic testing (Table 1).

**Table 1:** List of manuscripts reporting the use of umbilical cord blood for neonatal testing, along with brief description of findings

Laboratory Test	Author, year	Comments
CBC	Hansen, 2005 [5]	113 term infant paired umbilical cord & neonatal samples. High correlation of WBC, hematocrit, platelets. I:T ratio reported.
	Carroll, 2011 [3]	174 preterm infant umbilical cord & neonatal paired samples. High correlation of WBC, hemoglobin, platelets. False positive thrombocytopenia on 4 cord samples.
	Christensen, 2011 [4]	10 VLBW infant pilot study. No difference in CBC compared to historical controls.
	Beeram, 2012 [2]	200 term and preterm infant umbilical cord & neonatal paired samples. Similar results between sources. Higher rate of leukopenia in cord samples noted.
	Baer, 2013 [10]	91 VLBW infants. No paired samples. Outcome study.
	Rotshenker-Olshinka, 2014 [6]	305 term and preterm infant umbilical cord & neonatal paired samples. Significant correlation between cord and infant CBC.
Blood Culture	Polin, 1981 [8]	200 cord blood cultures with 29 paired infant cultures. Cord sterilized with 2 % tincture of iodine. One septic patient had positive cord and infant culture. Similar contamination rate between cord samples (2.5 %) and infant samples (3.4 %)
	Herson, 1998 [7]	81 term infants, 35 high risk umbilical cord & neonatal paired samples. Placental vein sterilized with povidone-iodine. 5 mL blood samples from cord obtained. 20 % true positive from cord vs. 3 % true positive from infant cultures of paired samples.
	Hansen, 2005 [5]	113 term infants. Cord sterilized with alcohol. Zero contaminants or true positive cultures
	Christensen, 2011 [4]	10 VLBW infant pilot study. No positive cultures from cases or historical controls.
	Beeram, 2012 [2]	200 term and preterm umbilical cord & neonatal paired samples. Cord sterilized with povidone-iodine and then swabbed with alcohol. 2 contaminants from cord blood. One contaminant and one pathogen from infant blood.
	Baer, 2013 [10]	91 VLBW infants. Prospective outcome study with historical controls.
	Rotshenker-Olshinka, 2014 [6]	223 term and preterm infant umbilical cord & neonatal paired samples. Cord sterilized with chlorhexidine prior to placental delivery. No cases of sepsis. High contamination rate from both cord sample (12.5 %) and infant sample (2.5 %).
Blood Type	AAP, 2004 [13]	Recommends “direct antibody test, blood type, and Rh (D) type on the infant’s (cord) blood.”
	Judd, 2001 [12]	Practice guideline for immunohematology. Endorses obtaining blood type from cord blood or infant blood.
	Christensen, 2011 [4]	10 VLBW infant pilot prospective outcome study with historical controls.
	Baer, 2013 [10]	91 VLBW infants. Prospective outcome study with historical controls.
Antibody Screen	Josephson, 2011 [13]	American Association of Blood Banking technical manual. Recommends antibody testing using plasma or serum from the infant or mother.
	Christensen, 2011 [4]	10 VLBW infant pilot study.
	Baer, 2013 [10]	91 VLBW infants. No paired samples. Outcome study.
Newborn Metabolic Screen	Miller, 2008 [14]	CLSI Newborn screening guidelines. Recommends first newborn metabolic test be obtained upon admission of premature infants.
	Christensen, 2011 [4]	10 VLBW infant pilot study.
	Baer, 2013 [10]	91 VLBW infants. No paired samples. Outcome study.

In paired-samples of cord blood versus blood drawn at birth from the neonate, CBC and leukocyte differentials are clinically equivalent. This has been demonstrated in both term and preterm infants [2–6]. In one study there was a small but notable subset of umbilical cord blood samples that exhibited a false positive thrombocytopenia result suggesting that when the platelet count is low from a CBC drawn from the cord, it should be confirmed with direct neonatal draw [3]. Another study reported a higher rate of leukopenia in cord blood samples compared to neonatal samples [2]. Together these studies produce compelling evidence that umbilical cord blood can safely be used in place of direct neonatal blood draw for a CBC with leukocyte differential. Repeat direct testing from the neonate may be needed in rare instances.

Several studies have contrasted blood culture results when blood is drawn directly from the neonate versus otherwise discarded blood from the umbilical cord or fetal vessels on the placenta [2, 4–8]. It has been noted that using otherwise discarded fetal blood offers the benefit of inoculating much higher blood culture volumes, which increases the sensitivity of the blood culture [7–9]. Concern is sometimes raised about contamination rates of umbilical cord blood cultures, particularly after vaginal birth. It is instructive to remember that the neonate is delivered through the same environment as the umbilical cord and placenta and care must be given to adequately sterilize the field prior to obtaining blood cultures, regardless of the anatomical site from which they are drawn. Several methods have been reported to adequately achieve a sterile cord/placenta including use of alcohol, betadine, and tincture of iodine. Because the surface of the placenta is often wet and contains depressions between vessels which allow for pooling of fluid it is beneficial to dry the surface prior to initiation of sterilization techniques. This procedure may not be necessary when a cord segment is used for obtaining a cord blood sample. With compelling data endorsing DCC or MUC cord, it should be noted that an adequate cord blood volume can be obtained even after either DCC or MUC [10].

Determination of the neonatal blood type is frequently done from umbilical cord blood. Both pediatric [11] and obstetric [12] literature specifically identify cord blood as an appropriate source for determining infant blood type and Rh (D) status. We are unaware of any studies that have specifically endorsed or refuted this practice for purposes of neonatal transfusion. It is our opinion that cord blood can be typed for the purpose of neonatal transfusion provided the sample is obtained from the umbilical cord and not from a fetal vein on the placental surface. Although the blood in the fetal vein on the placental surface is fetal blood, obtaining a sample in this manner introduces the possibility of operator error in which the needle is inserted through the fetal vessel and into the maternal chorionic villus space with resultant aspiration of maternal blood cells. Antibody screening for the purpose of transfusion of a neonate can also be performed on umbilical cord blood. The American Association of Blood Banking states, “initial patient testing must include . . . a screen for unexpected red cell antibodies, using either plasma or serum from the infant or mother” [13]. Further studies are required to further understand the optimal timing of obtaining cord blood and potential risks of this strategy.

Many states recommend drawing the newborn metabolic screening tests of VLBW infants before antibiotics are started, before blood products are transfused, and before amino acid-containing hyperalimentation solutions are administered. The Clinical and Laboratory Standards Institute recommends obtaining the first newborn metabolic screen upon admission for premature infants [14]. Obtaining this first sample from umbilical cord blood will result in similar limitations as an admission sample obtained directly from the infant. Specifically, despite being reliable for hemoglobinopathies, GALT and biotinidase enzymes as well as providing baseline testing for amino acids and acylcarnitines, admission testing may result in inaccurate TSH, 17-OHP, and cystic fibrosis (IRT) screening. In both scenarios repeat testing is required.

To date one pilot study [4] and one multicenter study [10] have been published reporting neonatal outcomes as a result of using umbilical cord blood for the admission laboratory testing in premature infants. The initial pilot study reported the outcomes of ten patients compared with historical matched controls. A decrease in erythrocyte transfusions and intraventricular hemorrhage was observed in the study patients. The larger multicenter trial enrolled 96 VLBW patients and successfully obtained umbilical cord blood for admission laboratory testing in 91. Cord blood was successfully obtained in twins, triplets, following DCC, and in eight of nine infants born at 23–24 weeks gestation. An increase in hemoglobin in the first 12–24 h of life, fewer transfusions per patient, fewer patients requiring any transfusion, and lower vasopressor use was observed in study patients. The rate of intraventricular hemorrhage appeared lower but was not statistically significant. To date no randomized controlled trials have been reported.

### Delayed cord clamping or umbilical cord milking at preterm delivery

**Fig. 1:**
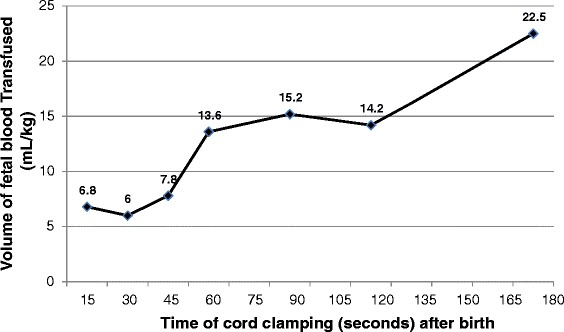
Amount of blood “transfused” from the placenta to the fetus during delayed cord clamping at various time intervals compared to early cord clamping at 5 s. Modified with permission from [41]

Studies over 45 years ago demonstrated a higher fetal blood volume and a proportionately lower placental blood volume in term infants as a result of DCC for up to 180 s [15]. At term birth, Yao et al. demonstrated a correlation between the time to cord clamping and infant blood volume. Using an iodinated albumin dilatation technique Yao demonstrated that delaying cord clamping by 30, 60, or 180 s increased the infant blood volume by 6, 14, or 23 mL/kg (Fig. 1). In 2011 Farrar et al. more clearly defined the volume and duration of placental transfusion [16]. This study provided a more detailed understanding of DCC by placing term infants on a scale with weight measured every 2 s until the umbilical cord was clamped. The authors reported a mean placental transfusion volume of 110 mL (32 mL/kg) following DCC. This volume of placental transfusion was significantly higher than previously reported. They also noted that although placental flow appeared to cease at 2 min for most infants it continued for up to 5 min in some. A recent multicenter trial by Vain et al. demonstrated similar results using serial weighing of neonates in order to estimate the volume of fetal blood transfused [17]. This study demonstrated variability between centers, with one reporting 8 mL/kg and another reporting 21 mL/kg following two minutes of DCC. The position of the infant relative to the mother, in both vaginal and cesarean delivery, was generally believed to influence the volume of fetal blood transfused by DCC [18, 19]. However, the recent randomized controlled trial by Vain et al. showed equivalent volumes of fetal blood delivered to the infant by DCC independent of infant position relative to the mother during DCC in term infants born by vaginal delivery [17].

For DCC to effectively transfuse placental blood to the newly delivered neonate fetal blood in the placenta must continue to transfer into the neonate for a period of time, i.e., potentially until the umbilical cord is clamped or cord pulsations cease. If the umbilical vein closes before the umbilical arteries, the neonate could “hemorrhage” back into the placenta. To study this, the direction of blood flow during DCC was evaluated using doppler ultrasonography in term infants after vaginal delivery [20]. The duration of flow in the umbilical vein and both umbilical arteries varied among the 30 infants in this study. Ninety percent had venous flow toward the neonate noted immediately after birth, while the remaining 10 % demonstrated no blood flow. Venous flow continued until the cord was clamped in 10/30 patients. Venous flow was bidirectional, with the direction of flow associated with the respiratory pattern. In some cases retrograde umbilical cord flow was observed during vigorous crying. It was previously postulated that umbilical arterial flow ceases quickly after delivery [21]. However, in this series of term infants flow continued until the cord was clamped in 13/30 patients. In half of the infants, the umbilical arterial and venous flow ceased at the same time, and in seven of the 30, arterial flow stopped before venous flow. In eight infants, venous flow stopped before cessation of arterial flow [22]. This study did not quantify flow rate or ratios of flow into the neonate versus flow from the neonate back to the placenta in cases where both arterial and venous flow continued. However, it provides important data demonstrating the variability of placental transfusion among infants and under certain physiologic conditions such as crying. Similar studies in premature infants are needed to identify the direction of flow and factors influencing placental transfusion in this population following both vaginal and caesarean delivery.

**Fig. 2:**
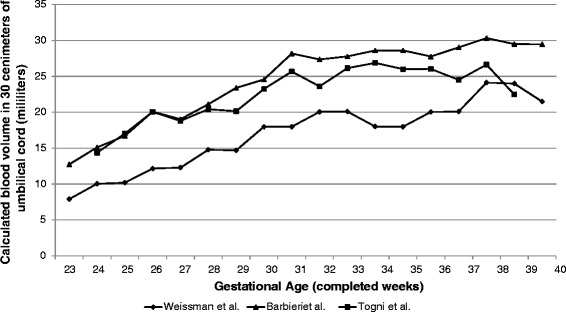
In-utero volume of blood contained within a 30 cm segment of umbilical cord at various gestational ages. Volumes were calculated from umbilical vein and artery diameters reported in the obstetric literature [23–25]

Milking of the umbilical cord is another method of transferring fetal blood from the placenta into the fetus before clamping and cutting the umbilical cord. This method offers the potential advantage of being completed more quickly than DCC. It may also be more enthusiastically adopted by obstetricians if they are uncomfortable holding an extremely premature infant during DCC without any perceived intervention. Several studies have used sonography to assess the umbilical vessel sizes at various gestational ages [23–25]. We used the reported umbilical vein and umbilical artery diameters to estimate the blood volume within the umbilical cord of a fetus (Fig. 2). This suggests that a 30 cm segment of umbilical cord may contain 8 to 28 mL of blood in a fetus at 23–34 weeks gestation. However, these values are highly speculative because they represent the diameter of umbilical vessels in utero rather than ex utero immediately after delivery. Hosono et al. recently sought to measure the actual amount of blood delivered by MUC [26]. Studying 20 ELBW neonates he found that a 30 cm segment of umbilical cord contained 15.5 ± 6.7 mL of fetal blood. Although this study may have overestimated the cord blood volume since the proximal and distal ends of the cord were not clamped simultaneously, it is the first study to report blood volume in a specific length of umbilical cord of preterm infants and thus provides valuable information.

Until recently, current practice was ICC following delivery of a premature infant. Over the past several years a number of organizations including the World Health Organization, Royal College of Obstetricians & Gynaecologists, and American College of Obstetricians & Gynecologists have formally endorsed DCC as standard of care [27–29]. The World Health Organization strongly recommends waiting at least one minute after birth before clamping the umbilical cord in term or preterm babies who do not require positive-pressure ventilation. The Royal College of Obstetricians & Gynaecologists supports DCC in healthy term infants with the infant not elevated higher than the mother’s chest prior to umbilical cord clamping. Their recommendations for preterm births suggest potential benefit by DCC and recommend development of strategies to provide initial neonatal care and resuscitation at the woman’s bedside with the cord intact. MUC is mentioned as an alternative but not formally endorsed as routine practice. The American College of Obstetricians & Gynecologists support DCC in preterm infants when feasible.

Premature infant brains are sensitive to fluctuations in blood pressure, particularly hypotension due to poor cerebral autoregulation [30]. Maintaining stable blood pressure may be a factor in limiting intraventricular hemorrhage [31]. Recently, Katheria et al. demonstrated improved superior vena cava blood flow and right ventricular output, both markers of systemic blood flow, in preterm infants following MUC compared to ICC [32]. Additionally, the diastolic and mean blood pressure within the first six hours of life was increased in the MUC group. This finding is consistent with previous studies on MUC [33, 34].

Recently benefits of DCC versus ICC have been assessed in premature infants. By 2004 at least seven randomized controlled trials compared ICC with DCC of preterm infants. Although the definition for DCC varied from 30 to 120 s, DCC was associated with higher hematocrits, fewer transfusions, and less intraventricular hemorrhage [35]. While some investigators were studying DCC others were testing MUC as a potentially more rapid alternative [33, 34]. In 2012, a Cochrane review included 15 randomized controlled trials on DCC up to 180 s or MUC, versus ICC [36]. The summary findings included 39 % fewer erythrocyte transfusions for anemia (7 studies), 41 % fewer patients with intraventricular hemorrhage (12 studies), and 38 % fewer patients with necrotizing enterocolitis (four studies). Although there was a 37 % decrease in death reported, this finding was not statistically significant. However, when analysis was limited to infants less than 32 weeks gestation [37], improved mortality rate was statistically significant. Backes et al. demonstrated that among infants <32 weeks undergoing either DCC or MUC, the mortality rate was decreased by 58 % (8 studies) [37]. With nearly 4 million births in the United States [38] and 1.93 % born before 32 weeks gestation [39] this technique could result in 4,500 lives saved annually in the United States with an even greater impact when worldwide births are considered. A lower mortality rate was independently reported in a manuscript of a quality improvement initiative following the implementation of MUC in all infants <30 weeks gestation [40]. Compared to historical controls the rate of survival after MUC increased from 84 to 94 % in neonates less than 30 weeks gestation, and increased from 76 to 91 % among infants less than 27 weeks gestation. Researchers continue to investigate the optimal duration of DCC, the best methods for MUC, and the best rational for using one versus the other technique in various clinical situations.

## Conclusion

Umbilical cord blood may be the most valuable underutilized resource in the care of premature neonates. Utilization of umbilical cord blood for admission laboratory testing of neonates is a promising new practice that has been shown to improve neonatal outcomes. While DCC and MUC have been formally endorsed by a number of professional organizations they have not yet been universally adopted by obstetricians and neonatologists. Full implementation of this practice is therefore an important step in better utilization of umbilical cord blood in improving the outcomes of premature neonates.

## Abbreviations

ELBW: Extremely low birth weight
VLBW: Very low birth weight
NICU: Neonatal intensive care unit
I/T ratio: Immature to total neutrophil ratio
DCC: Delayed cord clamping
MUC: Milking of the umbilical cord
